# Post-COVID-19-pandemic changes and clinical characteristics of invasive group a streptococcal infections from 2015 to 2023

**DOI:** 10.1007/s15010-024-02413-8

**Published:** 2024-10-17

**Authors:** Markos K. Tomidis Chatzimanouil, Susann Rößler, Dennis Nurjadi, Isidoros Iakovidis, Reinhard Berner, Nicole Toepfner, the Dresden G. A. S. Study Group: Stefan Richard Bornstein, Roland Aschoff, Martin Bornhäuser, Andreas Güldner, Florian Gunzer, Johannes Herold, Jurek Schultz, Pauline Wimberger, Thomas Zahnert

**Affiliations:** 1 Department of Pediatrics, Faculty of Medicine and University Hospital Carl Gustav Carus, TUD Dresden University of Technology, Fetscherstraße 74, 01307 Dresden, Germany; 2 Institute for Medical Microbiology and Virology, Faculty of Medicine and University Hospital Carl Gustav Carus, TUD Dresden University of Technology, Dresden, Germany; 3 Clinical Infectious Diseases and Antibiotic Stewardship Unit, Carl Carus University Hospital, Dresden, Germany; 4 Institute of Medical Microbiology and Clinic for Infectious Diseases, University of Lübeck and University Medical Center Schleswig-Holstein Campus Lübeck, Lübeck, Germany; 5 German Center for Infection Research (DZIF), Partner Site Hamburg-Lübeck-Borstel-Riems, Lübeck, Germany; 6 Department of Mathematics, University of Bologna, Bologna, Italy; 7 Department of Internal Medicine III, University Hospital Carl Gustav Carus, TUD Dresden University of Technology, Dresden, Germany; 8 Department of Dermatology, University Hospital Carl Gustav Carus, TUD Dresden University of Technology, Dresden, Germany; 9 Department of Internal Medicine I, University Hospital Carl Gustav Carus, TUD Dresden University of Technology, Dresden, Germany; 10 Department of Anesthesiology and Intensive Care Medicine, University Hospital Carl Gustav Carus, TUD Dresden University of Technology, Dresden, Germany; 11 Trauma and Plastic Surgery, University Center of Orthopaedic, University Hospital Carl Gustav Carus, TUD Dresden University of Technology, Dresden, Germany; 12 Department of Pediatric Surgery, University Hospital Carl Gustav Carus, TUD Dresden University of Technology, Dresden, Germany; 13 Department of Gynecology and Obstetrics, Technische Universität Dresden, Dresden, Germany; 14 Department of Otorhinolaryngology, Head and Neck Surgery, Carl Gustav Carus Faculty of Medicine, TUD Dresden University of Technology, Dresden, Germany

**Keywords:** Streptococcus pyogenes, Invasive GAS, Sepsis, Toxic shock, Clindamycin

## Abstract

**Purpose:**

Since winter 2022, invasive GAS (iGAS) infections have re-emerged in Europe, causing severe diseases in children and adults. We aimed to examine whether this reported post-pandemic increase was associated with an increased disease severity and/or a shift in clinical disease phenotypes.

**Methods:**

We performed detailed clinical phenotyping of patients hospitalized with iGAS infections at a 1410-bed tertiary German Medical Center from 01/2015 to 09/2023.

**Results:**

One hundred seventy-eight patients were included: 50 children (28.1%) and 128 adults (71.9%). IGAS infections of Q1/2023 exceeded the pre-pandemic average by 551% (1200% for children). The mean age of affected patients shifted significantly post-pandemically (49.5 ± 26.5 to 32.4 ± 28.2 years of age, p < 0.05), mainly due to the higher percentage of children affected with iGAS infections (15.2% pre-pandemic, 44.2% post-pandemic). CFR was significantly lower for children (2%) compared to adults (11.7%) (p < 0.05) and decreased from 13% to 6.5% post-pandemically (p = 0.148). Duration of antibiotic therapy (13.5 (10 to 21) to 10 (9 to 14) days), length of hospital (10 (4 to 25) to 7 (5 to 15) days), and ICU stay (7.0 (2.5 to 18.0) to 5.0 (3.0 to 8.5) days) were shorter post-pandemically. Despite the higher post-pandemic percentage of affected children, PICU admissions (57% before to 32% after), use of catecholamines (28.6% to 11.8%), invasive ventilation (35.7% to 17.6%) and CFR (7% to 0%) were all lower after the pandemic.

**Conclusion:**

Children were at higher risk for iGAS infections post-pandemically. The surge of post-pandemic iGAS infections was not accompanied by increased iGAS-associated morbidity and mortality.

**Supplementary Information:**

The online version contains supplementary material available at 10.1007/s15010-024-02413-8.

## Introduction

*Streptococcus pyogenes,* Group A Streptococcus (GAS), causes various infections in both children and adults worldwide. An increased incidence of invasive GAS (iGAS) infections and scarlet fever was reported by the World Health Organization (WHO) in 2022 for several European countries, including France, Ireland, the Netherlands, Sweden, the United Kingdom (UK), and Northern Ireland. IGAS infections affected especially children under 10 years of age, particularly during the second half of the year [1]. In the UK, a surge of iGAS infections was reported, particularly in those younger than 15 years [2]. In the Netherlands, an even higher increase for children aged 0 to 5 years [3] was revealed during a more than twofold increase of the annual average of iGAS in 2022 compared to pre-COVID-19 pandemic years. In spring 2023, the Robert Koch Institute and the German Reference Center for Streptococci noted a similar rise in Germany. A notable aspect of the iGAS infection surge was the spread of a more aggressive *emm1* GAS variant [4]. This variant was first identified in the UK (GAS M1UK) [4] and has subsequently been reported across Europe [5–7]. The M protein encoded by the *emm* gene is a main virulence factor of GAS often used for phylogenetic typing [8]. Along with GAS M1UK, another M1 GAS variant, M1DK, was recently discovered and has been rapidly expanding throughout Denmark [9].

Typically, national surveillance programs report on iGAS infections. These reports are often restricted to basic clinical information, such as age, gender, isolation source, diagnosis, and, in some cases, corresponding GAS M genotypes. GAS is a very versatile pathogen with numerous disease manifestations ranging from mild local throat [10] and skin infections to invasive local and systemic diseases [11] and post-streptococcal immune sequela. Besides genetic profiling of the pathogen [12], detailed clinical information on the affected patient group, infection characteristics, disease course, and treatment effects are essential to evaluate the impact of the current iGAS upsurge. We were interested in whether, along with the increased frequency of iGAS and the potential emergence of altered iGAS strains, the clinical characteristics of iGAS infections and affected patients also changed.

Therefore, the primary study aim was to examine whether the reported post-pandemic increase in iGAS infections in Germany was associated at our study site with an increased disease severity and/or a shift in clinical disease phenotypes. The secondary study aim was to identify differences in streptococcal disease courses between children and adults. The tertiary study aim was to determine if treatment with clindamycin and/or intravenous immunoglobulins (IVIG) decreased the case fatality rate (CFR) in streptococcal sepsis and streptococcal toxic shock syndrome (STSS).

## Methods

### Study design

The study was conducted as a retrospective single-centre cohort study at the University Hospital Carl Gustav Carus, TUD Dresden University of Technology, a 1.410-bed tertiary medical care hospital in Saxony, Germany. Inclusion criteria were as follows: patients at any age, hospitalized from January 1, 2015, to September 30, 2023, with an iGAS infection in any of the hospital’s departments. During clinical routine at the Institute for Microbiology and Hygiene, TUD Dresden University of Technology, all beta-hemolytic streptococci were isolated from blood agar (biomerieux Deutschland GmbH, Nürtingen, Germany) cultures. Isolates were identified as *S. pyogenes* via Matrix-assisted laser desorption/ionization time-of-flight mass spectrometry (MALDI-TOF MS, Bruker Daltonics GmbH & Co, Bremen, Germany). Strains isolated from blood, cerebrospinal fluid, pleural, abscess or joint aspirate, intraoperative bone biopsy or swab, and intraoperative intraabdominal swab were defined as invasive, and the respective patients were included in this study. For one patient, GAS detection in a pleural effusion was confirmed through 16rRNA PCR rather than culture. In accordance with local ethic regulations and after approval by the directors of all participating departments, patient histories of all detected cases were retrospectively assessed from the primary patient documents following a study case report form (CRF). Exclusion criteria were data from patients with non-iGAS infections and data from patients whose treatment of the acute iGAS infection had not yet been completed.

### Definition of study periods

Pre-, intra-, and post-pandemic study intervals were defined based on the first COVID-19- case detection and consecutive COVID-19 pandemic protection measures in Germany, as they affected the study site. These protection measures started on March 18, 2020, and ended on April 2, 2022. Accordingly, the pre-pandemic period spanned from quartile 1 (Q1, January to March), 2015 to Q1, 2020. The intra-pandemic period lasted from quartile 2 (Q2, April to June) 2020 to Q1 2022, and the post-pandemic period from Q2, 2022 to Q3, 2023. Differences between invasive streptococcal infections in the pre-, intra-, and post-pandemic study periods were analyzed.

### Case report form

In the study CRF the following data were recorded: age, gender, year and month of detection of the iGAS infection, diagnosis, immune status (competent/suppressed), prior local GAS infection, clinical symptoms and manifestation, maximal white blood count (WBC) (10^9/L), C-reactive protein (CRP) (mg/L), procalcitonin (PCT) (ng/mL), duration of antibiotic therapy, treatment with clindamycin and/or immunoglobulins, intensive care unit (ICU) or pediatric intensive care unit (PICU) admission, length of ICU/PICU stay, invasive and/or non-invasive ventilation, use of catecholamines, length of stay in the hospital and outcome.

### Study definitions of invasive GAS diagnosis

IGAS infections were divided into the following categories: Bacteriemia, sepsis, septic shock, STSS, pneumonia and parapneumonic effusions, osteomyelitis/septic arthritis, soft tissue infection (e.g., abscess, erysipelas, myositis, phlegmon, necrotizing fasciitis), meningitis, mastoiditis, and others (e.g. appendicitis, peritonitis). A patient could be assigned in more than one category, e. g., a septic patient with erysipelas and septic arthritis was assigned to soft tissue infection, osteomyelitis/septic arthritis, and sepsis. Regarding sepsis, septic shock, and STSS, each patient was assigned to only one category in ascending order of severity, i.e., a patient with septic shock was not assigned to sepsis but to septic shock. Similarly, a patient qualifying for STSS was neither assigned to sepsis nor septic shock but STSS.

Bacteriemia in both children and adults was defined as a positive blood culture without criteria fulfilment for sepsis, septic shock or STSS. Sepsis in adults was defined as an acute life-threatening organ dysfunction caused by an inadequate host response to an infection. The Sequential Organ Failure Assessment (SOFA) score was used to diagnose sepsis-associated organ dysfunction [13]. Septic shock was defined as arterial hypotension that persists despite adequate volume therapy, requires therapy with vasopressors to achieve a mean arterial blood pressure of ≥ 65 mmHg, while at the same time lactate level in the serum is > 2 mmol/l [13]. Based on the guidelines of the Surviving Sepsis Campaign [14] and other published data [15, 16], sepsis-associated organ dysfunction in children was defined as severe infection leading to organ dysfunction. Septic shock in children was defined as severe infection leading to cardiovascular dysfunction, including hypotension, need for treatment with a vasoactive medication, or impaired vascular perfusion. The Pediatric Sequential Organ Failure Assessment Score (pSOFA) was used to identify organ dysfunction in children [17]. STSS was defined as iGAS infection with hypotension plus multi-organ involvement, as defined from the Centres of Disease Control and Prevention (CDC) [18] and others [19, 20].

### Clindamycin and IVIG analyses

To assess the effect of clindamycin and/or IVIG administration in the CFR, we analyzed all patients with STSS and separately all patients with GAS septic phenotypes (sepsis, septic shock and STSS). Clindamycin and/or IVIG administration was accepted either as an empirical treatment or if administered after iGAS detection. All patients analyzed received empirically one or more doses of β-lactam antibiotic and had clindamycin susceptible isolates.

## Statistical analysis

Data features were assessed for normal distribution via the Kolmogorov–Smirnov test. Descriptive statistics are presented as mean ± standard deviation (SD) for normally distributed data or median (interquartile range (IQR)) if the variable did not fit normal distribution. Qualitative data are presented as n (%). Risk ratio (RR) was reported for categorical variables with a 95% confidence interval (95% CI). For continuous variables, *p* values are presented. Comparisons for normally distributed data were made using t-test and z-test for proportions, while a non-parametric approach was used for non-normally distributed data using the Mann–Whitney test. Post hoc Bonferroni correction was used for multiple comparisons and referred to as *p*_Bonf_. A value of *p* < 0.05 was considered to indicate statistical significance. All experiments and statistical analyses were performed with Microsoft Excel (Version 2309 Build 16.0.16827.20166) and the software Python (https://www.python.org/), with the packages SciPy (scipy.stats) and pandas.

## Results

### Study cohort

In total, 178 patients met the study inclusion criteria; 50 (28.1%) of them were children and 128 (71.9%) adults (Flow Chart). For 3 patients (2 children and 1 adult), GAS isolation in a sterile site was confirmed in hospitals other than the study site prior to transfer. The mean age of study patients was 42.2 ± 28.6 years (5.5 ± 3.9 for children (< 18 years of age) and 56.5 ± 19.9 for adults (≥ 18 years of age)). Similar gender distributions were observed in the pediatric (female 21 (42.0%) of 50, male 29 (58.0%) of 50) and adult (female 56 (43.8%) of 128, male 72 (56.3%) of 128) groups. Most patients were immunocompetent (155 (87.1%) of 178, 48 (96.0%) of 50 children, 107 (83.6%) of 128 adults). Only for a small percentage of patients local GAS infections (tonsillitis, impetigo, pyoderma) were reported prior to the invasive streptococcal infection (14 (7.9%) of 178 for the whole study cohort, 5 (10.0%) of 50 for children, 9 (7.0%) of 128 for adults).

### Seasonal trends of invasive streptococcal infections and post-pandemic increase

**Fig. 1:**
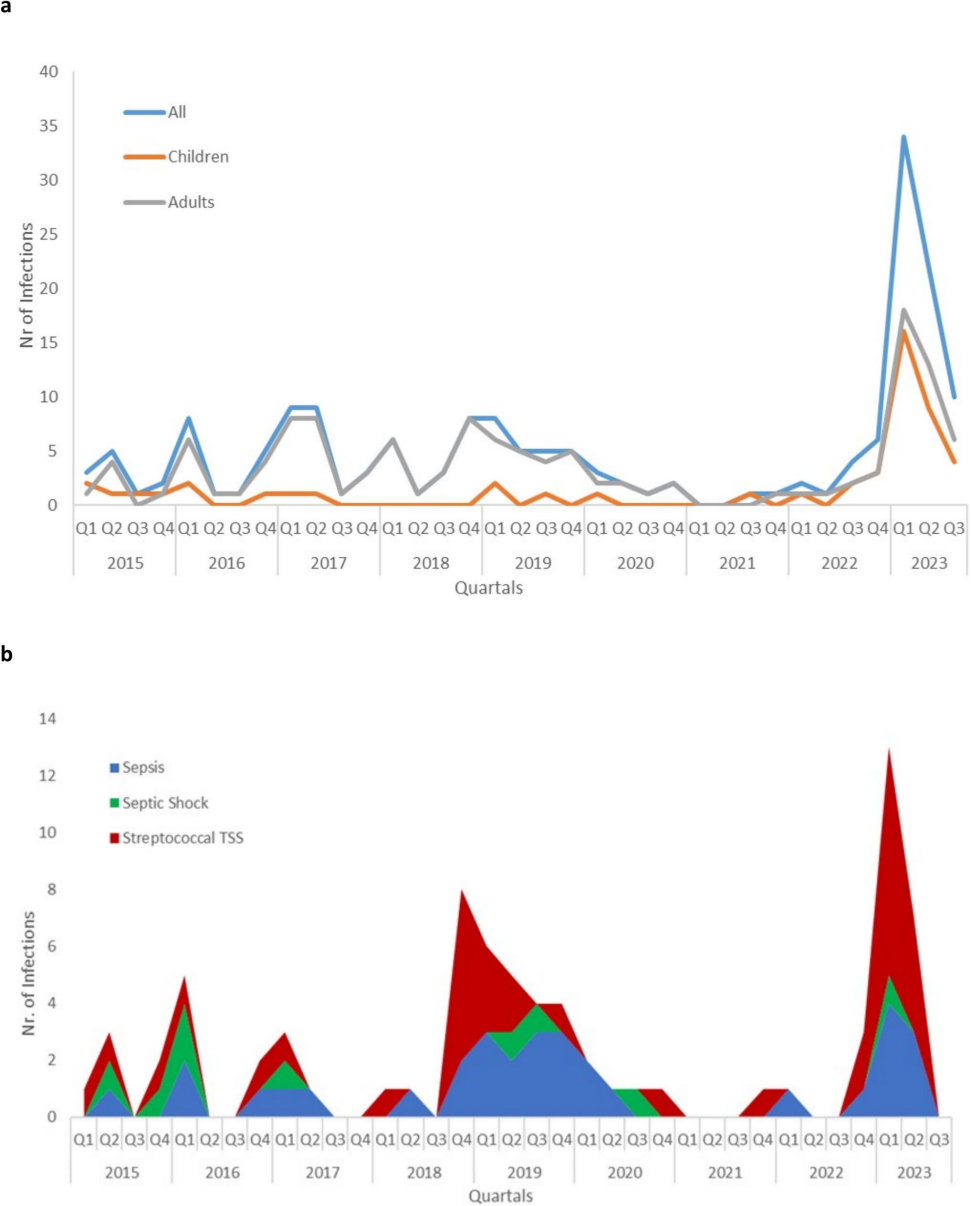
**a** IGAS infections at the University Hospital Carl Gustav Carus, TUD Dresden University of Technology from 2015 to September of 2023, presented in quartiles. **b** IGAS infections with septic phenotypes (sepsis, septic shock and STSS) from 2015 to September 2023 for both adults and children

From Q1/2015 to Q1/2020, the peaks of iGAS infections were detected predominantly in the first quarter of each year (Fig. 1a). From Q2/2020 to Q1/2022, iGAS infections decreased. Beginning from Q2/2022 and especially in Q1/2023 and Q2/2023, a strong increase in iGAS infections was detected. The average number of invasive infections in Q1, representing the seasonal peak from 2015 to 2020, was 6.2 ± 2.6, while the number of iGAS infections in Q1/2023 was 34, exceeding the previous average by 551%. This was especially the case for pediatric iGAS infections, where a 1200% increase (Q1/2023: 16, mean Q1 2015–2020: 1.3 ± 0.8) was observed, while a milder increase of 372% was detected for adults (Q1/2023: 18, mean Q1/2015–2020: 4.8 ± 2.7). Along with the increase of all iGAS infections, septic phenotypes increased by 433% (Fig. 1b) (Q1/2023: 13, mean Q1/2015–2020: 3 ± 2.1) and among them an even stronger increase of 686% was observed for STSS (Q1/2023: 8, mean Q1/2015–2022: 1.2 ± 1).

### Clinical spectrum of iGAS infections and post-pandemic changes

Table 1 shows the distribution of clinical phenotypes for iGAS infections before and after the pandemic. Soft tissue infections (66.3%) represented the bulk of iGAS infections before the pandemic. Septic disease phenotypes, including sepsis, septic shock, and STSS, comprised combined more than half of all iGAS infections (52.2%). Pneumonias accounted for 14.1% of infections, with less than half of them (6 of 13, 46.1%) accompanied with parapneumonic effusions.

**Table 1:** Distribution of clinical phenotypes for iGAS infections before and after the COVID-19-pandemic. A patient could score points in more than one category

	Pre-pandemic N = 92	Post-pandemic N = 77	RR (95% CI)
Bacteriemia	5 (5.4%)	5 (6.5%)	1.20 (0.36–4.01)
Sepsis	22 (23.9%)	8 (10.4%)	0.43 (0.20–0.97)
Septic Shock	7 (7.6%)	1 (1.3%)	0.17 (0.04–0.73)
Streptococcal TSS	19 (20.7%)	14 (18.2%)	0.88 (0.52–1.51)
All septic	48 (52.2%)	23 (29.9%)	0.57 (0.38–0.86)
Pneumonia, of which:	13 (14.1%)	12 (15.6%)	1.10 (0.50–2.45)
Parapneumonic Effusion	6 (6.5%)	10 (13.0%)	1.99 (0.65–6.13)
Osteomyelitis/ Septic Arthritis	17 (18.5%)	6 (7.8%)	0.42 (0.19–0.92)
Soft Tissue Infection	61 (66.3%)	47 (61.0%)	0.92 (0.70–1.21)
Meningitis	2 (2.2%)	4 (5.2%)	2.39 (0.47–12.18)
Mastoiditis	3 (3.3%)	8 (10.4%)	1.59 (0.48–5.34)
Others	9 (9.8%)	8 (10.4%)	1.06 (0.47–2.39)

Comparing the pre- to the post-pandemic era, the percentage of STSS remained at the same levels (20.7% to 18.2%), while there was a decrease of both sepsis and septic shock (10.4% and 1.3%, respectively). Altogether, septic disease phenotypes were lower in frequency (29.9%). Soft tissue infections remained (61.0%) first, while pneumonias where more likely to present with parapneumonic effusions (83.3%).

### Comparison between children and adult patients

A significant shift towards a younger age of affected patients was observed in the post-pandemic period compared to the pre-pandemic period (mean age of 49.5 ± 26.5 pre-pandemic, 32.4 ± 28.2 post-pandemic, *p* < 0.0001, *p*_Bonf_ < 0.001). In contrast, no significant differences could be observed when comparing the pediatric (4.6 ± 4.9 pre-pandemic, 6.0 ± 3.5 post-pandemic, *p* = 0.269) and the adult population (57.6 ± 19.8 pre-pandemic, 53.3 ± 20.3 post-pandemic, *p* = 0.259) separately. The percentage of children affected with iGAS infections was higher after the pandemic (14 (15.2%) of 92 pre-pandemic, 34 (44.2%) of 77 post-pandemic). Table 2 shows a comparison of clinical disease courses of iGAS infections between children and adults for the whole timeline examined.

**Table 2:** Comparison of disease courses for children and adults for the whole timeline

	Children (n = 50)	Adults (n = 128)	RR (95% CI) / *p* value
Case fatality rate	1 (2.0%)	18 (11.7%)	7.03 (1.61–30.68)
Intensive care unit admissions	20 (40.0%)	46 (36%)	0.90 (0.58–1.40)
Catecholamines	8.0 (16.0%)	34 (26.6%)	1.66 (0.92–3.01)
Invasive ventilation	12 (24.0%)	24 (18.8%)	0.78 (0.44–1.38)
Non-invasive ventilation	7 (14.0%)	8 (6.3%)	0.45 (0.17–1.16)
Length of intensive care stay (Days)	3.0 (2.0 to 8.0)	6.5 (3.0 to 18.0)	0.027
Length of hospital stay (Days)	8.0 (5.0 to 14.0)	10 (4.0 to 23.0)	0.391
Duration of antibiotics (Days)	10.0 (10.0 to 14.0)	10 (9 to 19.8)	0.982
Max. white blood cell count (in 10^9/L)	21.4 ± 10.8	18.2 ± 8.5	0.042
Max. C-reactive protein count (in mg/L)	156.6 ± 110.7	241.5 ± 134.1	0.0002
Max. Procalcitonin count (in ng/mL)	17.6 (1.0 to 27.1)	10.8 (1.8 to 47.5)	1

### Clinical course of iGAS infections and post-pandemic changes

Table 3 shows a detailed comparison of the post-pandemic changes in disease course of iGAS infections for the whole cohort and for the pediatric and adult cohorts separately.

**Table 3:** Post-pandemic changes in disease course of iGAS infections in children, adults, and the whole cohort

	Children		Adults		Total		
	Pre-pandemic (n = 14)	Post-pandemic (n = 34)	Pre-pandemic (n = 78)	Post-pandemic (n = 43)	Pre-pandemic (n = 92)	Post-pandemic (n = 77)	RR (95% CI)/ *p* value
Case fatality rate	1 (7%)	0 (0%)	11 (14.1%)	5 (11.6%)	12 (13%)	5 (6.5%)	0.50 (0.22 to 1.13)
Intesive care unit admissions	8 (57%)	11 (32%)	27 (35%)	16 (37%)	35 (38%)	27 (35%)	0.92 (0.59 to 1.43)
Catecholamines	4 (28.6%)	4 (11.8%)	18 (23.1%)	14 (32.6%)	22 (23.9%)	18 (23.4%)	0.98 (0.56 to 1.71)
Invasive ventilation	5 (35.7%)	6 (17.6%)	14 (17.9%)	8 (18.6%)	19 (20.7%)	14 (18.2%)	0.88 (0.48 to 1.61)
Non-invasive ventilation	2 (14.3%)	5 (14.7%)	5 (6.4%)	3 (7.0%)	7 (7.6%)	8 (10.4%)	1.36 (0.50 to 3.76)
Length of intensive care stay (Days)	2.5 (1.8 to 8.0)	4.0 (2.5 to 8.0)	8.0 (4.0 to 21.0)	5.0 (3.0 to 9.3)	7.0 (2.5 to 18.0)	5.0 (3.0 to 8.5)	0.362
Length of hospital stay (Days)	9.0 (5.0 to 17.3)	8.0 (6.0 to 14.0)	10.0 (4.0 to 27.0)	7.0 (5.0 to 21.8)	10 (4 to 25)	7 (5 to 15)	0.309
Duration of antibiotics (Days)	14.0 (10.0 to 14.0)	10.0 (10.0 to 14.0)	13.0 (10.0 to 21.0)	10.0 (7.0 to 17.0)	13.5 (10 to 21)	10 (9 to 14)	0.071
Max. white blood cell count (in 10^9/L)	22.1 ± 12.8	21.1. ± 10.0	18.0 ± 9.2	19.1 ± 7.1	18.6 ± 9.9	19.9 ± 8.4	0.370
Max. C-reactive protein count (in mg/L)	173.4 ± 87.6	149.6 ± 122.2	247.5 ± 131.4	236.8 ± 143.7	236.8 ± 128.3	200.4 ± 141.0	0.081
Max. procalcitonin count (in ng/mL)	33.9 (25.2 to 131.2)	10.5 (0.7 to 17.6)	10.0 (2.3 to 65.9)	15.7 (0.8 to 39.5)	12.2 (2.8 to 65.9)	12.0 (0.7 to 30.1)	0.224

### Case fatality rate

CFR of all iGAS infections was 10.7% (19/178), with CFR of 25.0% (19/76) for sepsis and 40.0% (14/35) for STSS. CFR ranged from 13% pre-pandemically to 6.5% post-pandemically (*p* = 0.148) *(*Table 3*)*. The CFR for pediatric patients was significantly lower (2%) compared to adults (11.7%) for the whole study period (*p* = 0.005) *(*Table 2*)*.

### Treatment of iGAS sepsis and STSS

Clindamycin was administered to 19 (54.3%) of 35 STSS patients. The CFR was 56.3% (9/16) for STSS patients without clindamycin and 26.3% (5/19) for STSS patients with clindamycin administration (*p* = 0.06). When considering all septic iGAS infections, clindamycin was administered to 32 (42.1%) of 76 patients. The CFR in septic patients was 31.8% (14/44) without clindamycin administration and 15.6% (5/32) with clindamycin administration (*p* = 0.085). Immunoglobulins were administered to 9 (25.7%) of the 35 STSS patients. No difference between treatment with or without immunoglobulins was observed regarding the CFR (44.4% with immunoglobulins to 38.5% without, *p* = 0.757). When considering all septic iGAS infections, immunoglobulins were administered to 10 (15.2%) of 76 patients. The CFR was 40% without and 22.7% with administration of immunoglobulins (*p* = 0.289).

## Discussion

The WHO has reported a post-pandemic increase of iGAS infections for many European countries [21–23], including France, Ireland, the Netherlands, Sweden, the UK, and Northern Ireland. This increase mostly affected children under ten and was particularly marked during the second half of 2022 [1].

Together with the report of the Robert Koch-Institute, Berlin, and the National German Reference Center for Streptococci, Aachen, the findings of the analysis presented here support a relevant increase in iGAS infections in Germany [24]. Our study illustrates the post-pandemic iGAS effect in a German tertiary care hospital, with an increase of 1200% in pediatric and 372% in adult patients. The reasons for this post-pandemic burst of iGAS infections are manifold and have not yet been clearly unraveled. One reason could be a change in molecular virulence patterns of the invasive GAS strains, as the current re-emergence was associated with a spread of a new emm1UK GAS clone [4–6, 25–29]. Host susceptibility for iGAS infections could be an additional or independent factor contributing to the enormous post-pandemic increase. A reduced mucosal and antibody-based immunity against GAS due to pandemic containment measures, as well as less contact with GAS as throat commensals and to GAS skin and throat infections, are discussed. Additionally, a transient compromised immune response following COVID-19 infection is suggested but remains elusive [30, 31].

Regarding the distribution of the reported iGAS infections, soft tissue infections were the most frequent pre- (66%) and post-pandemically (61%). After the pandemic, the rate of septic phenotypes (including sepsis, septic shock, and STSS) increased by 433%, with a general increase of 551% for all iGAS infections. The distribution of septic diseases among all iGAS infections, however, lowered from more than half of infection (52%) pre- to 30% post-pandemically. In comparison, parapneumonic effusions caused by GAS doubled and contributed to GAS-related morbidity from 6.5% to 13%. The rate of STSS remained similar for both periods (20.7% vs 18.2%).

In line with other reports [1–3], the current re-emergence of iGAS infections predominantly affected children and young adolescents, with almost half of the patients (44.2%) being children in the post-pandemic period compared to only 15.2% in pre-pandemic years. This led to a considerable shift in the age of all affected iGAS patients (mean age of 49.5 ± 26.5 pre-pandemic, 32.4 ± 28.2 post-pandemic, *p* < 0.0001, *p*_Bonf_ < 0.001). Comparison of the two groups revealed a significantly lower CFR for pediatric patients compared to that of adults (2% vs. 11.7%, *p* = 0.005). Additionally, a trend towards a shorter length of stay in the hospital and fewer days in the ICU was observed in pediatric compared to adult patients.

When comparing the clinical disease courses in the post- with the pre-pandemic period, ICU admissions and treatment during the ICU stay regarding catecholamines, ventilation, and laboratory inflammation markers showed little variation. In fact, a trend towards a shorter length of stay in the hospital, shorter duration of antibiotic therapy, and lower CFR was evident post-pandemically. This also holds true for the pediatric study cohort alone, with no evidence for increased severity of iGAS infections in the post-pandemic period for any of the above parameters. Interestingly, despite the higher post-pandemic percentage of affected children (15.2% of all iGAS infection before the pandemic to 44.2% after), the percentage of PICU admissions (57% before to 32% after), the use of catecholamines (28.6% to 11.8%), invasive ventilation (35.7% to 17.6%) and the CFR (7% to 0%) were all lower after the pandemic. These findings are in line with a recent study [32] reporting that the course of iGAS disease in children and adolescents in 2022–23 was not more severe than in previous seasons.

Importantly, similar results were also shown for the adult cohort, where age did not significantly differ pre- and post-pandemically (57.6 ± 19.8 pre-pandemic, 53.3 ± 20.3 post-pandemic,* p* = 0.259). Only the use of vasopressors increased after the COVID-19 pandemic. At the same time, the CFR, the duration of ICU and hospital stay, and the duration of antibiotic therapies were lower in the post-pandemic period, with ICU admissions and invasive ventilation showing little variance. From a clinical point of view, it is important to confirm that the clinical manifestations of iGAS infections did not aggravate during the pre-/post-pandemic increase. In absolute numbers, fatal outcomes might occur more frequently, but this is due to the increase in iGAS infections rather than to a more fatal variant of the pathogen. In comparison to adults, iGAS infections increased especially in children. Infections in this group were potentially less severe due to the younger age and less comorbidities.

This retrospective analysis supports the findings of previous studies [33, 34] that additional treatment with clindamycin in sepsis, septic shock, and STSS caused by GAS could be beneficial. In our study, additional administration of clindamycin reduced the CFR by half in the STSS cohort (56.3% to 26.3%) as well as in the cohort of all septic phenotypes (31.8% to 15.6%), although the results did not reach statistical significance (*p* = 0.06). The additional benefit of clindamycin has been attributed to the prevention of GAS toxin production as it acts as a bacteriostatic drug down-regulating ribosomal protein translation [35]. In German [36] and other guidelines [18, 37–39], clindamycin is recommended for both pediatric and adult patients with STSS in combination with high-dose penicillin. Similar to previous studies [33], the results of the present study support the administration of clindamycin not only for STSS but for all septic iGAS infections.

The effect of IVIG on STSS has been discussed in some recent studies [34, 40]. In this study, IVIG had no significant beneficial effect. The contradictory trend indicating that the administration of immunoglobulins was associated with higher fatality in GAS sepsis and STSS patients was most probably because IVIG were applied as an ultima ratio in the most severely affected patients.

The main limitation of this study is the retrospective study design in a single center. Retrospective studies can hide significant flaws, such as data omission or recall bias. However, primary patient data were available for all patients included in this study, and data acquisition was realized in a systematic manner. Additionally, we were able to perform a longitudinal observation spanning eight years. Another limitation is that the groups comparing efficacy of clindamycin and immunoglobulins are heterogeneous, since no matched pair case–control study could be conducted due to the retrospective nature of the study, and that in only 10 of 76 septic cases immunoglobulins were administered.

In summary, the findings of this study revealed a strong post-pandemic increase of iGAS infection in a single center in Germany, but iGAS-associated morbidity and mortality did not increase. Pediatric patients were post-pandemically at a higher risk for iGAS infections. Additional clindamycin treatment was beneficial not only in patients with STSS but also in patients with all septic manifestations of iGAS diseases.

## Supplementary Information

Below is the link to the electronic supplementary material.

Supplementary file1 (DOC 59 KB)

## Data Availability

The author confirms that all data generated or analysed during this study are included in this published article. Furthermore, primary sources for this study are available from the authors upon reasonable request.

## Abbreviations

GAS: Group A Streptococcus
iGAS infection: Invasive Group A Streptococcus infection
STSS: Streptococcal toxic shock syndrome
WHO: World Health Organization
UK: United Kingdom
TU: Technical University
CRF: Case report form
WBC: White blood count
CRP: C-reactive Protein
PCT: Procalcitonin
RR: Risk ratio
CI: Confidence interval
PICU: Pediatric intensive care unit
ICU: Intensive care unit
IVIG: Intravenous Immunoglobulin
CFR: Case fatality rate
