# Supplementary material for: Post-COVID-19-pandemic changes and clinical characteristics of invasive group a streptococcal infections from 2015 to 2023
Source: Infection. 2024 Oct 17;53(3):991–1000. doi: 10.1007/s15010-024-02413-8 (PMC12137492; doi:10.1007/s15010-024-02413-8)
Supplement: Supplementary file 1 — Supplementary file1 (DOC 59 KB) [file 15010_2024_2413_MOESM1_ESM.doc]

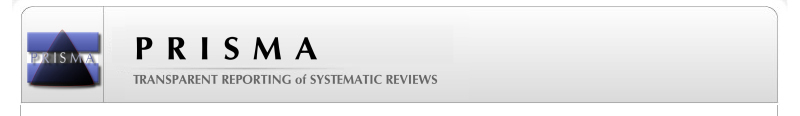
**PRISMA 2009 Flow Diagram:** Post-COVID-19-pandemic changes and clinical characteristics of invasive group A streptococcal infections from 2015 to 2023

**Screening**

**Included**

**Eligibility**

**Identification**

Study cases identified through GAS detection in a primary sterile body sites at the microbiological department
(n = 371)

Additional study cases identified through other sources
(n = 0)

Study cases after removal of duplicates (due to > 1 invasive GAS isolate per patient (n=170))
(n = 201)

Records analyzed
(n = 201)

Full patient records assessed for eligibility
(n = 201)

Study cases excluded due to isolation of GAS from non-strictly sterile site
(n = 23)

Records included in analysis (n = 178) from following departments:

Pediatrics (including PICU): 38

Pediatric Surgery: 10

Internal Medicine: 27

Intensive Care Medicine: 33

Orthopedics: 23

ENT: 32 (including 2 pediatric patients)

Gynecology: 4

Dermatology: 11
